# Hospital and Institutional News

**Published:** 1916-09-02

**Authors:** 


### HOSPITAL AND INSTITUTIONAL NEWS.

### "THE HOSPITAL'S" EDUCATIONAL NUMBER.

At the beginning of the academic year in September we publish as usual a Special Educational Number, to enable intending medical students and those responsible for deciding the medical career of young men and women to decide in the light of expert knowledge how to determine their choice among the various branches of the profession. An account of the new field of appointments which continues to unfold in medicine is always difficult for the layman to find, and this year as usual The Hospital covers a different branch from that dealt with in preceding years. A special article is devoted to the prospects of Dental Surgery, in the light of which its advantages and drawbacks are clearly explained. The opportunities offered to medical officers in the Navy are described; and the future of Public Health Work is discussed in a third article. Those now having to exercise their choice are often in doubt as to the effect of the war on the recent expansion of Public Health appointments, and therefore it becomes desirable to clear up the prospects ahead. These and other articles on medical careers of the established kind are supplemented by one on the position of Research Work in this country, which more advanced students and intending specialists will find reviewed both as at present organised and with suggestions for its coordination and endowment as a definite department of medicine. Full details are added as to the curricula and fees of the Medical Schools.

### THE LATE SIR R. B. MARTIN AND THE SUNDAY FUND.

The death of Sir Richard Biddulph Martin, of the historic banking house which bore the family name, removes one of the early sponsors of the Metropolitan Hospital Sunday Fund; and a link with the past is broken with his death. We quote his personal recollections of the inception of the fund from the *Morning Post*: "The scheme," he is reported to have stated, "was suggested in Martin's office, and some of us held a little meeting at the London Tavern, at which we resolved to start the Fund that is now so well known. The real author of the suggestion was Mr. Ramsey, who was secretary of St. Mark's Hospital. Ramsey himself derived it from Canon Miller, of Birmingham, who some time before had founded just such a fund in that city. We went to the Lord Mayor of the day, Sir Sydney Waterlow, who was very sympathetic, and with whose aid the fund was started." Sir Sydney communicated with the Mayor of Birmingham, Alderman Ambrose Biggs, asking him to obtain full particulars of the Hospital Sunday organisation in Birmingham. He asked Mr. Henry Burdett to prepare a full statement of the facts, which the latter did. This was forwarded to Sir Sydney at the Mansion House, and with the active co-operation of the *Lancet* Hospital Sunday was instituted in London in 1873. Martin's Bank thus adds to its own venerable associations that of possessing the room in which the Metropolitan Hospital Sunday Fund was, not conceived, but hatched. Presumably the old secretary's room at St. Mark's Hospital has the earlier honour, so far as the Metropolitan Fund was concerned. Sir Richard Martin was one of the pleasantest and kindest of men. He was devoted to the interests of the Metropolitan Hospital Sunday Fund and has proved a tower of strength to St. Mark's Hospital throughout his life. His many friends will sincerely regret his death.

### SUDDEN DEATH OF A HOSPITAL CHAIRMAN.

As we go to press we learn that Major A. J. Pell, D.L., J.P., Chairman of the General Committee of Addenbrooke's Hospital, died suddenly at Tattenhall, near Chester, on Monday evening, at the age of fifty-three. Major Pell had held the chairmanship for twelve years, during which time important improvements have been effected in the system of government; whilst much additional accommodation for patients has been provided in the shape of wards for children and a new outpatient department; a new clinical laboratory has also been added. Major Pell took a deep interest in these extensions and improvements, and it is not too much to say that the position of Addenbrooke's Hospital to-day is much stronger than when he first took office. Major Pell, who was Chairman of the Fulbourn Asylum Committee, and Chairman of the Quarter Sessions for the Isle of Ely, offered his services to the War Office on the outbreak of war as musketry instructor, and a few months ago was appointed Musketry Staff Officer with one of the divisions of the New Army. We cannot do full justice here to Major Pell and his work, but shall hope to find another opportunity of doing so.

### THE RESULTS OF WOUND TREATMENT IN MILITARY HOSPITALS.

At the opening by Lord Islington the other day of an addition to the military section of the Great Northern Central Hospital in Holloway Road, Lieut.-Colonel Callender, officer commanding the 2nd London General Hospital, made the statement that of those patients who have been treated at the Great Northern (a constituent part of the 2nd London General) since the beginning of the war 75 per cent. have recovered sufficiently to return to the Service. This interesting statement must not be taken as an estimate applying to the whole of the wounded in military hospitals; the recovery rate as a whole is rightly kept secret by the military censorship. But it does show that the Great Northern is doing first-rate work, and that in asking for an extension of its activities the authorities were paying the staff there a well-deserved compliment. The Germans have published on various occasions preposterous figures about their recovery rate, intended for consumption abroad, but too clumsily dishonest to deceive anyone with inside knowledge. One of the acutest of the war commentators has adopted a 60 per cent. standard of recoveries, and even that is perhaps too generous if by recovery is meant fitness for full active service in the front lines. The new building, now adapted to hospital purposes, has hitherto been the North Islington Library, Manor Gardens; it will provide eighty-two beds for wounded soldiers. The staff of the G.P.O. Money Order Department, which is next door, have contributed £120 and have named a ward. It is announced that anyone can name a bed for a contribution of fifteen shillings a week.

### A POINT FOR THE CAMBRIDGE MEDICAL SCHOOL.

Some revised instructions concerning the decoration of the Military Cross have just been issued by authority. In one small particular these new regulations affect one of the great medical schools, that of Cambridge University. They provide, that is to say, for the use of the initials M.C. after the name of any individual upon whom this decoration has been bestowed. Now these initials are already in use to denote those to whom Cambridge University has granted the degree of Master in Surgery. The number of surgeons thus affected is small, for the degree is very sparingly granted; but all the same there is certain to be occasional confusion. It is perhaps hard that the long priority of the University in respect of these letters should not entitle it to secure possession; but we do seriously suggest that it would be well to abandon "M.C." for Masters in Surgery, and to assimilate the practice of other Universities by changing either to M.Ch., or M.S., or C.M.

### ANTHRAX IN THE SHAVING-BRUSH.

Once more anthrax has been traced to an infected shaving-brush, this time at Newcastle. A case occurred in that city, investigation of which incriminated a cheap horsehair shaving-brush, imported from Japan. It turned out that this was one of a large consignment newly arrived from the East. Immediate steps were taken by the public health authorities to track down all the infected brushes, and it is believed that practically all of them have been recovered before further mischief had been disseminated. The brushes are described as having black japanned wooden handles and a thin brush of white hair. They sell for about 2d. each—another instance of the fatal mania for cheap nastiness in which the British nation has so long allowed itself to be educated. A high proportion of the brushes confiscated are stated to show the presence of anthrax germs.

### EAST AFRICA AND THE RED CROSS.

The unity of the Empire could hardly be better illustrated than by the folllowing notice which has been issued by the Secretary of State for the Colonies: "The Government of the East Africa Protectorate is contributing £500 to the collection which is being made in East Africa for the British Red Cross Society and Order of St. John, in return for the material assistance rendered by the Society in the local campaign." That the Red Cross should aid in every theatre of war is natural and obvious, but that the remoter Dependencies should transmit contributions to this country brings home to all of us the unity and interdependence of the free nations now fighting under the British flag.

### THE NEW HOSPITAL ON THE MERSEY.

This week another important military hospital is to be inaugurated with the conversion of the new Town Hall of Wallasey, only just completed, to hospital purposes. The building has cost £100,000 and provides accommodation for 350 beds. The site, on the Cheshire bank of the Mersey, is a fine one, and a medical and nursing staff are already understood to have been secured. In view of the Army Council's refusal of the offer of the Dewsbury Guardians to place the whole of their Union Infirmary, Staincliffe, at its disposal, it seems that the recent demand for largely increased hospital accommodation has been met. For, in explaining why this offer was declined, the Army Council stated that "the normal numbers of sick and wounded are amply provided for by the hospitals already formed," and that "the recent extension of hospital accommodation was intended to meet an emergency which has now been largely provided for." Wallasey Town Hall thus falls into line with a regular scheme that should, we hope, make the taking over of any more schools, at any rate, unnecessary.

### A SPECIAL HOSPITAL FOR CANADIANS.

In view of the plea for the further use of hotels as hospitals, additional interest is given to the opening of the Peak Hotel, Buxton, as the special hospital of the Canadian Red Cross Society. The opening ceremony was performed appropriately by the Duchess of Devonshire, wife of the Governor-General Designate of Canada. This latest unit of the Canadian Medical Services can accommodate nearly 300 men, and is intended for soldiers suffering from shell-shock and gunshot-wounds. The special treatment includes peat packs and mineral-water baths. The hospital is commanded by Major Guest, and the matron is Miss MacAllister, who has a staff of fifteen trained nurses. The above, of course, provides an obvious instance of the suitability of a hotel for conversion to hospital purposes, since it already possesses in the baths part of the equipment required.

### "HOUSED WORSE THAN CATTLE."

While the taking over of institutions for the purposes of military hospitals on the whole has tended to improve their sanitary condition, a deplorably reverse effect appears to have been produced in the case of many vessels commandeered for animal transport. According to Dr. Ernest A. Acomb, acting medical officer of health to the Newport Port Sanitary Authority, the attendants in many cases have been housed worse than the cattle. In his report, issued last week, Dr. Acomb declares that the proportion of vessels found to be defective in sanitation is higher now than it was in the previous year. In one instance, where alterations had been made to accommodate a larger number of Lascars, all the regulations were infringed. In the larger vessels taken over for the transport of horses and mules overcrowding and filthy quarters were found. In one vessel, a temporary structure, knocked up so badly as to admit both rain and spray, 15 feet by 30 feet in size, contained fourteen bunks. This was the men's only place to live, sleep, and keep their things in! The cattle decks were actually better, and some of the horse stalls were used by the men. The facts cited are their own criticism, and we hope that the report will be brought prominently before the authorities in charge of animal transport. When we add that the medical officer to the Port of London declares that the quarters of the crews in most merchant vessels are habitually overcrowded, the case for drastic reform is complete.

### DISABLED OFFICERS AT REGENT'S PARK

Some few weeks ago attention was drawn in these columns to the tendency for Regent's Park to become a centre for institutions devoted to blind, paralysed, and otherwise disabled officers and men. The latest development of that tendency is the decision to convert St. John's Lodge, Regent's Park, into a hospital for paralysed and disabled officers. The building has been lent by the Treasury and by the Office of Woods and Forests to the British Red Cross Society, and Sir John Ellerman has offered to defray the whole of the expenditure for a year or for such further time as may be agreed. The house will be ready for the reception of fifty patients in six or eight weeks' time, and it is to be called the Sir John Ellerman Hospital. As the care and treatment of disabled officers is one of the chief objects of the Kitchener Memorial Fund, the Council of that Fund has been invited to appoint representatives on the committee of the Sir John Ellerman Hospital, in order that advantage may be taken of the experience which will be gained in the treatment of such cases in the hospital. Arrangements have been made with the Royal Botanic Society for the upkeep of the gardens, the cost of which Sir John has also undertaken to bear.

### A "UNIFORM SYSTEM" IN INDIA.

In order to co-ordinate Red Cross work in India under one organisation, an Indian branch of the Joint Committee of the Red Cross Society and the Order of St. John was lately formed, with divisions in the various Presidencies. It is especially interesting to note that one of the first steps in this process of relating the new branches with each other and with the Central Committee in Pall Mall is a mission to standardise the system of accounts, for which purpose Mr. Harold M. Barton, F.C.A., has left for India and Mesopotamia. The standardisation of accounts, or, in other words, a Uniform System, in all branches of hospital work is the true preliminary to the prevention of waste, and we are glad to see this necessary step is being taken in the organisation of Red Cross work in India. The War Charities Act at home and this new undertaking in India should do much to place the Red Cross finances in every form as much above reproach under the Uniform System of Hospital Accounts as this system has achieved in the case of our voluntary civil hospitals.

### LARGE BEQUESTS BY THE CHAIRMAN OF THE BON MARCHE.

The charitable bequests of Mr. Edwin Jones, of Clapham Park, founder of the drapery firm of Jones and Higgins and chairman of the Brixton Bon Marché, aggregate a very large sum, though not one comparable with the enormous totals of the two estates mentioned in these columns in our last issue. Amongst other charities the following hospitals benefit at once: King's College Hospital, £500; the Royal Hospital for Incurables, Putney, £500; the Brompton Hospital for Consumption, £500; the Flintshire Dispensary and Cottage Hospital, Holywell, £500. Upon the death of his wife, even larger sums are bequeathed to hospitals and other charities. King's College Hospital then gets £2,000; the Brompton Hospital for Consumption, £1,000; the Royal Hospital for Incurables, Putney, £1,000 also. About nineteen bequests of smaller amounts are included in this disposal of the residual estate. The total sum immediately and eventually available for charity amounts to about £19,200; the gross value of the estate is sworn at £189,644, including personalty of the net value of £153,592.

### AN INSTITUTIONAL ABUSE VENTILATED IN THE DAILY PRESS.

It is to be hoped that the ventilation in the correspondence columns of the *Daily Mail* of the alleged practice of forbidding nurses to sit down while on duty in hospital wards will be the complete abolition of such a custom wherever it may exist. From the tenor of the correspondence it would seem that at some hospitals there is an unwritten rule, which nurses are afraid to break for fear of getting a reputation for slackness. Certainly no written rule to this effect exists, as far as our experience goes; if it does, it should at once be abolished. Unwritten rules, however, are more difficult to deal with than written ones in many cases: it should be the business of every hospital manager, whether secretary, superintendent, or committeeman, to ascertain whether such a custom prevails in his institution; and if it does to take the necessary steps at once both to abolish the custom and to let every nurse be fully and definitely aware that it is abolished. We feel sure no matron in her senses would allow or encourage such a custom if she knew of it. If any such there be they should be firmly dealt with by superior authority.

### WAR CONDITIONS IN SCOTTISH ASYLUMS.

Interesting details as to the effect of the war upon the General Board of Control for Scotland are given in that body's second annual report, which has been issued as a White Paper. For reasons of national economy the document is considerably reduced in size, many statistical tables having been omitted. No expenditure upon new buildings for the care of mental defectives has been undertaken since the outbreak of war, and work was also stopped in connection with the erection of asylums for the insane. The Edinburgh and Renfrew District Asylums have been converted into military hospitals—the former into an ordinary medical and surgical hospital, the latter into a special hospital for mental and nervous cases—and their entire staffs, medical and lay, are in military service. This arrangement would not have been possible but for the co-operation of the Royal Edinburgh Asylum, the Paisley District Asylum, and the Greenock Parochial Asylum, which consented to receive for the period of the war all new cases of mental deficiency or insanity from the Edinburgh and Renfrew districts. The report states that the extensive enlistment of male attendants into the Army, notwithstanding the greater employment of women in the nursing of male patients, is still giving rise to embarrassment. Many of the assistant matrons and nurses possessing hospital qualifications are absent on military service, and the duties and responsibilities of all those who remain at their posts have been greatly added to.

### THE ANTI-TUBERCULOSIS CAMPAIGN IN ABERDEEN.

The lack of adequate housing accommodation is, perhaps, the most serious drawback with which the would-be tuberculosis reformer finds himself confronted. Of what avail is the most approved form of sanatorium treatment if, after his discharge, the patient has to spend the remainder of his existence in some ill-ventilated bed-sitting room, the air-space of which is further reduced by other occupants and cumbrous furniture? So hopeless does the chance of recovery seem under such conditions that one is tempted to question the value of sanatorium treatment at all unless the after homelife can be modelled upon the same plan. Dr. Matthew Hay, the Medical Officer of Health for the City of Aberdeen, has pointed out in his recent annual report that the principle of rent grants to certain of the poorer households would enable the consumptive patient to be better separated from the rest of the family, and so would offer him a better chance of recovering his health sufficiently to enable him to resume his work. At a recent meeting of the Public Health Committee the proposal to allocate an annual sum of £100 to assist in the payment of the rent of the houses in which tuberculous patients were found, in order to help them to obtain better accommodation, was discussed. Dr. Hay estimates that about £5 annually would probably suffice for the needs of any one given case, and that the sum suggested would enable the Health Department to deal with twenty of the most urgent cases. It may be noted that the Health Committee resolved to recommend the Town Council to approve of the proposal as an experiment, and the successful carrying out will be watched by other authorities with great interest.

### A NEW SUBJECT FOR WAR HOSPITAL GAZETTES.

With the exception of two articles on Canada and Serbia, the August number of the *Norfolk War Hospital Magazine* is mainly composed of fiction and light verse. Its late appearance is explained by the departure of the editor, Dr. I. D. Eaton, for Malta, to the recent pressure of work, and, if we may attempt to interpret a cryptic phrase, to illness among the editorial staff. It should perhaps be explained that the sketches we have summarised as fiction are largely incidents connected with some aspect of the war at the Front or in hospital, which patients and others have contributed. The atmosphere is suitable to a war hospital magazine, and so long as a tale is a good tale only a very jejune reader will care whether it be a record of fact or not. The article on "Canada" leads us to wish that patients from the Dominions should be encouraged to contribute accounts of their own country to these publications. They would perform an equally interesting and careful work if they would also set down their impressions of England, for we should learn almost as much about Canada from a Canadian's description of our own ways as they appeared to him, as from a sketch of Canadian life. Such a series of articles would be eagerly read, and we hope that the present one on "Canada" may be followed up by others on the lines that we have suggested.

### THIS WEEK'S DRUG MARKET.

The drug market has been unusually quiet and almost uneventful during the past week. This is attributable to the same cause that has been in operation for some little time—namely, the weakening of prices, which has made buyers extremely cautious. Notwithstanding this steady downward tendency, actual alterations in quotations of a substantial kind are very few from week to week, but the easier tone is sufficiently pronounced to influence buyers to make their purchases fit in with their immediate requirements. After a long period of stagnation quinine has been in better demand, and a fair business has been done at rather lower prices; this refers to Continental brands of quinine, for British makers' quotations remain unaltered. Acetanilide is again rather lower, and it is quite probable that the lowest figure has not yet been reached. Aspirin also still has a slightly downward price tendency. On the other hand, barbitone is dearer owing to increasing scarcity, and as supplies of phenacetin are still insufficient to meet the demand the value of this drug has again advanced. Oil of bergamot is considerably dearer. The price of ipecacuanha is not so firmly maintained. Bromides are quiet and unchanged. At the recent public sale of drugs held in Mincing Lane there was an unusually poor demand, and the bulk of the offerings did not find buyers. Cape aloes, cardamoms, honey, kola-nuts, and senna-leaves all had an easier tendency in value.

